# A Safe-Domain Generative Adversarial Network with Swin Transformer for Noisy Imbalanced Fault Diagnosis

**DOI:** 10.3390/s26123754

**Published:** 2026-06-12

**Authors:** Xiao Lai, Xiaohan Zhang, Zhiqi Xie, Min Liu

**Affiliations:** 1 School of Electronic and Electrical Engineering, Shanghai University of Engineering Science, Shanghai 201620, China; 32260002@sues.edu.cn (X.L.); xieziqi@sues.edu.cn (Z.X.); 2 College of Computer Science and Technology, Tongji University, Shanghai 201804, China; 3 College of Electronic and Information Engineering, Tongji University, Shanghai 201804, China; zhangxiaohan@tongji.edu.cn

**Keywords:** fault diagnosis, imbalanced data, label noise, data generation, safe domain, Transformer

## Abstract

Currently, data-driven fault diagnosis methods have achieved remarkable progress. However, in industrial scenarios, acquiring a sufficient amount of fault data poses a challenge, thereby leading to the issue of imbalanced data in intelligent fault diagnosis. Furthermore, manual recording and instrument measurement errors will introduce label noise, which significantly impacts diagnosis performance. To address these problems, this paper proposes a safe-domain generative adversarial network with Swin Transformer (SDGAN-ST). A safe domain selection method is utilized to eliminate noisy samples and construct a pure dataset that poses no risk to the GAN training process. Consequently, GAN can generate high-quality minority samples to rebalance the original dataset. Additionally, the Swin Transformer is employed as a classifier to capture global information for each fault sample, thereby achieving high diagnostic accuracy. Experiments on the CWRU dataset and a real-world oxygen compressor bearing dataset demonstrate the effectiveness of the proposed method. On the CWRU dataset, SDGAN-ST achieves accuracies of 98.88%, 97.63%, and 97.50% under imbalance ratios of 1:10, 1:20, and 1:30, respectively. On the real-world dataset, SDGAN-ST achieves 100% accuracy under all three imbalance ratios. Additional experiments under noise ratios of 20%, 30%, and 40% show that SDGAN-ST maintains stable diagnostic performance and is more robust to label noise than ordinary WGAN-GP-based methods.

## 1. Introduction

Enhancing the overall competitiveness of the global manufacturing industry relies heavily on intelligent manufacturing, which has become a core technology [1,2]. However, due to the harsh conditions in which equipment often operates, such as high speed and pressure, mechanical components such as bearings are highly prone to damage [3]. The damage of a single small part can result the malfunctioning of the whole system. The current approaches for fault diagnosis can be categorized into two groups: the mechanism-driven and the data-driven methods [4]. The former are limited by the operational mechanism of equipment and lack learning capabilities, thus exhibiting poor generalization performance when dealing with massive data [5]. Alternatively, fault diagnosis with data-driven methods involves feature extraction from monitored data rather than relying on the development of mathematical models. Recently, some data-driven methods, especially those based on deep learning, have achieved satisfactory performance [6]. For example, Niu et al. [7] proposed a deep residual convolutional neural network (CNN) with an enhanced discriminate feature learning capability to diagnose bearing fault. In [8], a bidirectional long short-term memory network and a capsule network with CNN were combined for fault diagnosis. Tang et al. [9] utilized a Vision Transformer (ViT) for bearing fault diagnosis. Zhang et al. utilized time-series Transformer [10] and Mixure-of-Modality-Diagnosis Transformer [11] for electromechanical system fault diagnosis. Quan et al. [12] proposed a KPCA-ISSA-KELM model for fuel cell system fault diagnosis, in which KPCA was used for feature extraction and dimensionality reduction, and ISSA was adopted to optimize KELM. However, these methods heavily rely on a sufficient amount of data to achieve great performance. In real scenarios, the normal working condition of mechanical equipment predominates, making it challenging to collect adequate fault data. Consequently, fault diagnosis often encounters the problem of imbalanced data.

To tackle the aforementioned concern, researchers have employed various approaches to synthesize minority fault data with the objective of achieving balance between healthy and faulty samples. These methods can be categorized into two classes: sampling techniques [13,14] and deep-learning-based data generation models [15] like generative adversarial networks (GANs) [16,17,18,19,20]. In fact, researchers prefer utilizing GANs for data generation due to its ability to extract latent space features. For instance, Liu et al. [18] proposed a deep feature enhanced GANs to generate fault data for bearing imbalanced fault diagnosis. Similarly, Luo et al. [19] adopted a Wasserstein GAN with gradient penalty (WGAN-GP) to reconstruct a balanced dataset. In [20], the WGAN-GP and an oversampling technique were integrated by a dual-attention feature fusion network to generate bearing fault data. However, there still exists a limitation in the above methods: they all assume that the monitored data is pure and noiseless. But, in complex industrial conditions, the monitored data often contains label noise by the errors of instrument measurement and manual recording [21]. The presence of these noises significantly degrades the quality of the generated data, consequently undermining the precision of diagnosis.

Aiming at the noisy imbalanced fault diagnosis, we propose a novel model named Safe-domain GAN with Swin Transformer (SDGAN-ST). This model comprises three main components: a denoising module, an ordinary WGAN-GP, and a Swin Transformer based classifier. Specifically, we employ a safe domain selecting method to select relatively clean and noiseless samples. These selected samples form a safe domain set that serves as the training data for the WGAN-GP. The WGAN-GP then generates high-quality fault samples to balance the original dataset, addressing the issue of data imbalance. Finally, the Swin Transformer is utilized to provide accurate diagnostic results. This manuscript is an expanded version of our preliminary conference work [22]. Compared with the preliminary version, this study further explains the safe-domain selection mechanism, introduces a more advanced Swin-Transformer-based classifier, and newly adds a real-world oxygen compressor bearing dataset for practical validation.

The main contributions of this paper are as follows:

(1) A SDGAN is designed for imbalanced fault diagnosis with label noise. The model exhibits the capability to generate samples of exceptional quality even in the presence of label noise.

(2) We present a safe domain selecting method for determining the safe domain of each fault class. This method effectively mitigates label noise and facilitates the creation of a clean training set for subsequent data generation models.

(3) The Swin Transformer is employed as the classifier for fault classification and demonstrates superior diagnostic accuracy compared to both the ViT and traditional CNN methods.

## 2. Preliminaries

### 2.1. Noisy Imbalanced Fault Diagnosis

Deep learning models are highly dependent on sufficient data. However, in industrial scenarios, the faulty conditions are far less than the healthy. As a result, it is relatively easy to collect a large amount of data representing the healthy state, while data representing faulty conditions is extremely scarce. In the field of machine learning, when one class is significantly more dominant than another in the training set, it is referred to as an imbalanced dataset. Considering a dataset with *C* classes, denoted by labels (*y_1_*, …, *y_C_*). Supposing that the number of samples labeled by *y_a_* is denoted by *N_a_*, the number of samples in each class is (*N_1_*, …, *N_C_*). If there exists *N_i_* (*i* ∈ 1, 2, …, *M*) that significantly exceeds any *N_j_* (*j* ∈ *M* + 1, *M* + 2, …, *C*), the classes labeled as *y_i_* is considered as majority classes while those labeled as *y_j_* are regarded as minority classes. The imbalance ratio (IR) in this dataset can be computed using the following formula:

(1) $$IR=\frac{N_{j}}{N_{i}}\times 100\%$$

In classification tasks, when the training set is imbalanced, the resulting trained model tends to favor the frequent classes (i.e., majority classes). This bias occurs because the model aims to minimize errors mainly for those majority classes. As the severity of IR increases, the inclination towards frequent classes will also aggravate. The inclination arises from the fact that achieving higher overall accuracy is closely tied to correctly predicting the classes that occur more frequently in the dataset. Consequently, there is a significant risk that the fault diagnosis model may disregard the minority classes (i.e., fault classes), which is unacceptable in real scenarios.

In industrial conditions, data noise, particularly label noise, is a prevalent issue due to measurement errors commonly encountered in sensors. If the true label of a sample is *y_m_* but is mislabeled as *y_n_* (*m ≠ n*), this sample is considered as label noise. In this context, the noise ratio (NR) can be computed using the following formula:

(2) $$NR=\frac{N_{mis}}{N_{all}}\times 100\%$$

where the *N_mis_* is the number of mislabeled samples while the *N_all_* is that of all samples in a class. When encountering label noise in addition to imbalanced data, a fault diagnosis task is referred to as noisy imbalanced fault diagnosis. A huge amount of correctly labeled data is highly required for training deep learning models with the supervised learning theory that aim to minimize empirical error. If some label noise exists in the dataset, the computed empirical error does not accurately reflect the true error, resulting in a trained network that deviates further from achieving generalization error minimization. Therefore, the diagnosis model becomes susceptible to being misled by the label noise and acquiring incorrect knowledge, ultimately leading to lower diagnostic accuracy.

### 2.2. Wasserstein GAN with Gradient Penalty

The GAN is a common data generation model consisting of a generator and a discriminator. The generator takes a random noise vector as input and aims to generate output that as similar as possible to the real training samples. On the other hand, the discriminator’s role is to differentiate between the real samples and the generated samples produced by the generator, while the generator is to deceive the discriminator as much as possible. The two networks confront each other and learn from each other’s feedback. Finally, the discriminator will be unable to reliably determine whether a given sample is real or generated. Meanwhile, a powerful generator is obtained that can produce high-quality synthetic samples.

The original GAN utilized Jensen–Shannon (JS) divergence to measure the distance between real and generated sample distributions. However, due to its fixed value of log2 when these two distributions don’t intersect, JS divergence fails to accurately reflect the distance. As the distributions are unlikely to intersect at the beginning of training, the JS divergence remains constant. This can result in a zero gradient for the GAN, making it difficult to converge. To solve the issue, Arjovsky et al. [23] proposed the WGAN which employs Wasserstein distance to replace the JS divergence. The Wasserstein distance can be calculated as follows:

(3) $$W\left(P_{r},P_{g}\right)=\frac{1}{K}\underset{\|f{\|}_{L}\leq K}{\sup}E_{x\sim P_{r}}[f(x)]-E_{x\sim P_{g}}[f(x)]$$

where *P_r_* and *P_g_* are the distributions of the real and the generated samples, and *f* denote a K-Lipschitz continuous function. At this time, the discriminator aims to fit the function *f*.

To meet the Lipschitz constraint in (3), Gulrajani et al. [24] introduced the gradient penalty (GP) as a regular term and proposed the WGAN-GP. After the above improvement for the initial GAN, the WGAN-GP is more stable in the training process and easier to converge.

### 2.3. Swin Transformer

Swin Transformer [25] is a variant of the Transformer architecture and proposed by Microsoft Research Asia in 2021 for image recognition. Swin Transformer improves the multi-head self-attention (MSA) in ViT [26] as the window MSA (W-MAS) and shifted window MSA (SW-MSA) and introduces a hierarchical architecture to process high-resolution images with less computational complexity.

The Swin Transformer contains a patch partition module and four feature learning stages which consists of a linear layer (or patch merging process) and several Swin Transformer blocks. The pipeline can be summarized as follows: At first, an image is input to Swin Transformer and cut into many patches in the patch partition module. Then, in the first feature learning stage, a linear layer is used to compute the embedding for each patch and several Swin Transformer blocks are applied afterwards for feature extraction. In the other three stages, the patch merging process downsamples the patch number for the hierarchical representation at first, and the subsequent Swin Transformer blocks work the same as the first stage.

Each block is composed of the multi-layer perceptron (MLP), layer normalization (LN), W-MSA, and SW-MSA. The W-MSA is computed within a local window (the number of patches in each window is fixed). And the SW-MSA achieve information interaction across previous windows by shifting window, so that the Swin Transformer can maintain the global receptive field. The transformation of data can be formulated as follows:

(4) $$z_{l}^{\prime }=W\text{-}\mathrm{MSA}(\mathrm{LN}(z_{l-1}))+z_{l-1},l\in (1,2,\ldots ,L-1)$$

(5) $$z_{l}=\mathrm{MLP}(\mathrm{LN}(z_{l}^{\prime }))+z_{l}^{\prime },l\in (1,2,\ldots ,L-1)$$

(6) $$z_{l+1}^{\prime }=\mathrm{SW}\text{-}\mathrm{MSA}(\mathrm{LN}(z_{l}))+z_{l},l\in (1,2,\ldots ,L-1)$$

(7) $$z_{l+1}=\mathrm{MLP}(\mathrm{LN}(z_{l+1}^{\prime }))+z_{l+1}^{\prime },l\in (1,2,\ldots ,L-1)$$

where *z_l_* denotes the output of the *l*-th block.

## 3. The Proposed Diagnosis Method

To tackle the issue of noisy imbalanced fault diagnosis, a SDGAN-ST is proposed in this paper. This chapter begins by presenting a framework of our model in Section 3.1. In Section 3.2, we explain the method for selecting a safe domain and the composition of SDGAN. Finally, in Section 3.3, we introduce our classifier based on the Swin Transformer.

### 3.1. Framework

**Figure 1:**
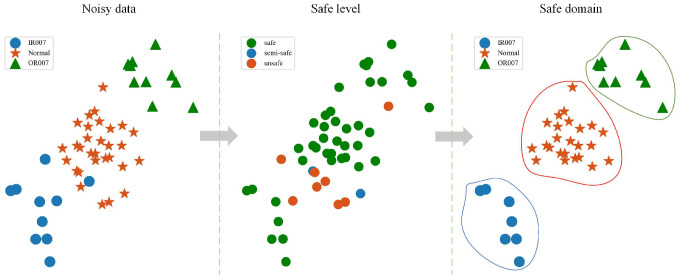
The visualization of selecting safe domains.

**Figure 2:**
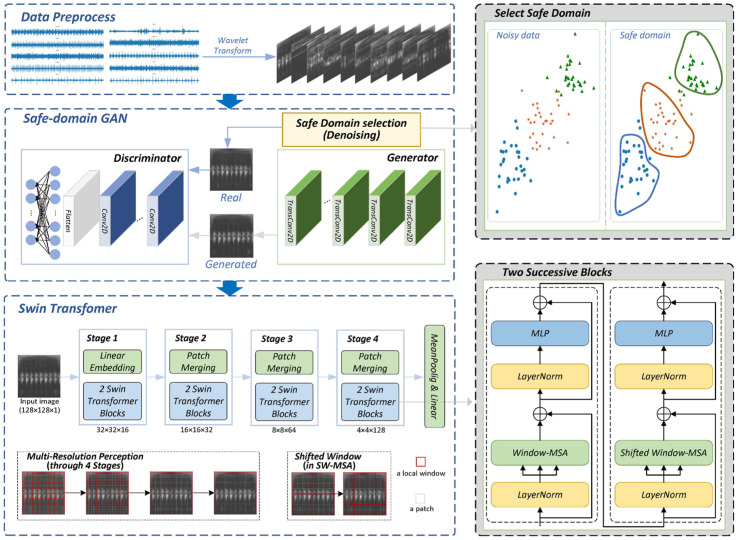
Framework of the proposed SDGAN-ST.

The whole fault diagnosis method consists of three modules: a wavelet transform (WT)-based data preprocessing module, a SDGAN-based data generation module, and a Swin-Transformer-based classifier. The steps for the imbalanced fault diagnosis with noisy data are as follows: First, the original 1D vibration signals are transformed to 2D time-frequency image using WT. Then, our SDGAN is adopted to mitigate the negative impact of noise through safe domain selection as illustrated in Figure 1, and generate fault samples in order to balance the initial dataset. Finally, we utilize a Swin Transformer for fault diagnosis. The framework of our method can be seen in Figure 2.

### 3.2. Safe-Domain GAN

To reduce the negative impact of noise, we developed a method to assess the risk for each sample and select a safe domain, where the samples are considered to be harmless to subsequent networks. The safe-domain selection method is inspired by the local-neighborhood idea of Safe-Level-SMOTE [11]. Both methods assume that the reliability of a sample can be estimated according to the label consistency of its neighboring samples. In Safe-Level-SMOTE, the safe level is mainly used to guide interpolation-based oversampling, so that new samples are generated in relatively safe regions. In contrast, the proposed method adapts this idea to GAN training. Instead of directly generating synthetic samples by interpolation, the proposed safe-domain selection method first identifies reliable samples and constructs a relatively clean subset for adversarial learning. The selected safe-domain samples are then used to train the WGAN-GP, while unsafe samples are excluded to reduce the negative influence of mislabeled data on the generator. Therefore, the novelty of the proposed safe-domain selection method lies in its role as a noise-aware sample-filtering mechanism for GAN-based fault sample generation, rather than as a direct oversampling strategy. The method procedures are as follows:

- Construct a set *D* containing all labeled samples.
- For each sample *p* in *D*, compute its *K* nearest neighbors, where the *K* is a predefined constant.
- If there is at least one neighbor with a different label from *p*, *p* is considered an unsafe sample. Otherwise, randomly select a sample, denoted as *q*, from the *K* neighbors of *p*. In this case, *p* is regarded as a candidate reliable sample, and the neighborhood of *q* is further examined to distinguish safe and semi-safe samples.
- Compute the *K* nearest neighbors of sample *q*.
- If there is at least one neighbor with a different label from *q*, the local neighborhood around (*p*) is not fully reliable, and, thus, *p* is defined as a semi-safe sample. Otherwise, *p* is defined as safe.
- After processing all samples in *D*, the safe level is attached to each sample.
- For each class *C*, calculate the proportions of safe, semi-safe, and unsafe samples among the total samples.
- If the combined proportion of unsafe and semi-safe samples exceeds 50%, the safe domain for that class includes both safe and semi-safe samples. Otherwise, the safe domain consists only of safe samples.

After applying the aforementioned selection method, we can obtain the safe domain for each class. This method offers two advantages: on the one hand, it can effectively eliminate mislabeled samples, ensuring the reliability of the dataset; on the other hand, the method strikes a balance by not excessively filtering out samples, which prevents the training set for the GAN from becoming too small in scale. This ensures that an adequate number of samples are retained for training while still maintaining the improved quality and reliability of the dataset.

The robustness of the safe-domain selection strategy comes from the local label-consistency assumption. In the feature space, correctly labeled samples are more likely to be surrounded by samples from the same class, whereas mislabeled samples usually show inconsistency with their local neighbors. Therefore, the proposed neighbor-based criterion can assign higher risk to samples located in inconsistent neighborhoods and prevent these samples from being used for GAN training.

We combine the safe domain selecting method and the WGAN-GP as the Safe-domain GAN. Following the procedures of the above selection method, we consider all the data together that have been preprocessed by WT and calculate the security domain of each class. And we then train the WGAN-GP with the data in safe domains. The fully trained generators are used to synthesize samples and rebalance the dataset.

The loss function of the discriminator is as follows:

(8) $$\begin{array}{cc} \underset{f}{\min}\;L_{D} & =E_{x\sim p_{r}}[f(x)]-E_{x\sim p_{g}}[f(x)]\\ & -\lambda E_{\hat{x}\sim p_{\hat{x}}}\left[{\left({\left\|\nabla_{\hat{x}}f(\hat{x})\right\|}_{2}-1\right)}^{2}\right] \end{array}$$

where the *f* represents the inference process of the discriminator, the *P_r_* and *P_g_* are the distributions of the real and generated separately, and the $\hat{x}$ can be calculated as follows:

(9) $$\hat{x}={\mathrm{tx}}_{r}+(1-t)x_{g}\;,\;t\in U\left[0,1\right]$$

which implies that $\hat{x}$ is the linear interpolation between the real data *x_r_* and generated data *x_g_*. On the other hand, the loss function of the generator is as follows:

(10) $$\underset{G}{\min}\;L_{G}=-E_{z\sim p_{z}}[f(G(z))]$$

where *G* is the inference process of the generator and *z* is the random noise input.

### 3.3. Swin-Transformer-Based Classifier

Inspired by the excellent performance of Swin Transformer in image recognition for its strong feature extraction ability, we introduced it as the classifier to recognize the fault modes. The Swin Transformer we adopted in this paper contains a patch partition module, four feature learning stages, a mean-pooling layer, and a MLP classification head. The details of structure are shown in Table 1.

The input grayscale image is resized at first. In the patch partition module, the image is divided into several patches with a small patch size. In the linear embedding layer of Stage 1, the raw-valued feature of each patch is projected to an embedding representation. Then, the patches are processed by two Swin Transformer blocks where the pixel number in a local window is few so that the network can capture the details of the image and obtain high-resolution features. In the patch merging layer of Stage 2, each group of four neighboring patches are combined as a new patch and then projected into a new representation by a linear layer. In other words, the patch merging layer achieves a 2× downsampling of resolution in order to obtain relatively low-resolution features. Then, two Swin Transformer blocks are used for feature extraction. The procedures of Stage 3 and 4 are the same as that of Stage 2.

After the computation of four stages, the hierarchical feature is obtained. For the image classification task at this section, we only need to focus on the topmost features. We add a mean-pooling layer and a MLP at the top to summarize the overall feature of the input and predict its class. We also add a Softmax function after Swin Transformer so that a vector is output as the probabilities that the input image belongs to each class respectively. The loss function of the Swin Transformer based classifier is cross-entropy, which can be calculated as follows:

(11) $$\underset{p}{\min}\;L=\sum_{i}l(i)\log(p(i))$$

where the *p* denotes the prediction of the Swin Transformer and *l* is the one-hot coding for the true label of the input *i*.

## 4. Case Study

To prove the effectiveness of the proposed diagnosis model, we designed some comparative experiments on the Case Western Reserve University (CWRU) dataset [27] and a real-world bearing dataset separately. All experiments are coded with the PyTorch 1.12.0 framework and run with an Intel Core i5-10400F CPU and a NVIDIA GeForce GTX 3090 GPU. In both cases, the hyperparameters of the SDGAN are same. Specifically, the *K* of the safe domain selecting method in the SDGAN is set as 3. This small value is adopted because the minority classes contain limited samples under high imbalance ratios, and a small neighborhood can better preserve local label consistency while reducing the risk of including samples from other classes. The learning rate of the SDGAN is 0.00005 and the epoch is 80,000. As for Swin Transformer, the structures of two cases both follow Table 1, but the epochs are set as 100 and 20 separately with the learning rate of 0.0001. The random seed is fixed during dataset splitting, noisy-sample generation, and fault classification to ensure reproducibility.

### 4.1. Case I: Experiments on CWRU Dataset

(1) Dataset Construction

The CWRU rolling bearing open dataset is a well-known dataset in the field of fault diagnosis. The dataset was collected through experiments conducted on a Reliance Electric motor, where faults were introduced into the motor bearings using Electro-discharge machining technology. The faults were created with diameters ranging from 0.007 inches to 0.040 inches and were located in the inner race, ball, and outer race of the bearings.

For this study, we specifically selected the bearing fault data from the drive end. The data was sampled at a frequency of 12KHz and a motor speed of 1797 RPM. The fault types in these data can be categorized based on their size, which include 0.007 mm, 0.014 mm, and 0.021 mm, as well as their location, which include inner race, ball, and outer race. By including the normal condition, we obtained a total of ten classes. To convert the vibration signals into a format suitable for analysis, we utilized the WT to transform the signals into 2D grayscale images with a resolution of 128 × 128. Specifically, continuous wavelet transform (CWT) with the Morlet mother wavelet is adopted to obtain the time-frequency coefficient matrix of each vibration segment. The obtained coefficient matrix is normalized to the range of [0, 255] and then resized to a grayscale image with a resolution of 128 × 128.

To simulate the label noise situation for each fault class, we randomly put some samples of other classes inside. For each target class, a given proportion of samples from the other classes is randomly selected and inserted into the training subset of the target class. These inserted samples keep their original signal contents but are treated as samples of the target class during training, thereby forming mislabeled samples. This process introduces symmetric class-mixing noise because the noisy samples can be randomly selected from all other classes. The validation and test sets keep their original labels and are not contaminated by label noise. Totally, we constructed three imbalanced datasets with the IRs of 1:10, 1:20, 1:30 separately and the NR of 20%. The construction details are shown in Table 2.

(2) Data Generation

**Figure 3:**
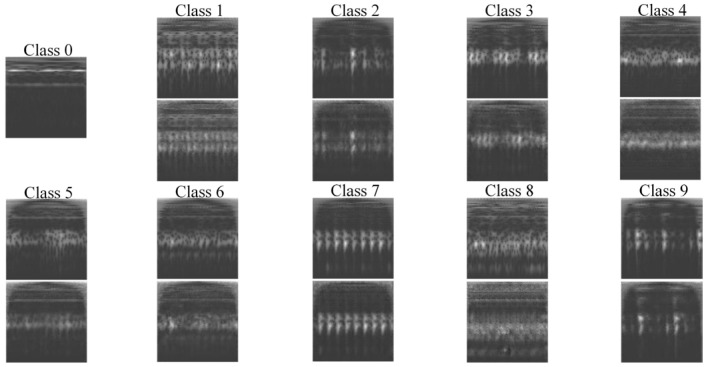
The data display of 10 classes of CWRU balanced dataset. In a pair of images arranged up and down in class 1–9, the top one is real and the bottom one is generated. The figure shows that the generated samples preserve similar time-frequency patterns to the real samples.

For each fault class, the safe samples are selected first, and then used for the training of GAN. Finally, the trained generator is used to synthesize samples which will be combined with the original real samples to balance the dataset. For example, we select 24 safe samples of class 1 in the IR of 1:10 to train the generator. And 270 images were generated and combined with the original 30 images to get a total of 300 images. The procedure is repeated for the other classes, and then a balanced 10-class dataset is obtained. The real and generated images of nine fault classes in the IR of 1:10 are shown in Figure 3. It can be seen that the overall quality of the generated image is very high.

(3) Noisy Imbalanced Fault Diagnosis

As our ultimate concern is the imbalanced fault diagnosis performance of our model, we compare the diagnostic accuracies of various methods in different IRs. We also used a CNN and a ViT as the other classifiers to compare with the Swin Transformer. This CNN consisted of five convolutional layers and two fully connected layers. And the ViT retains only the encoder side which contains four Transformer blocks with the model dimension of 256. For the no-generation baseline, the classifier is trained directly on the original imbalanced training set without any generated samples. To ensure a fair comparison, the no-generation baseline uses the same classifier architecture, optimizer, learning rate, batch size, and number of training epochs as the corresponding generation-based methods.

**Figure 4:**
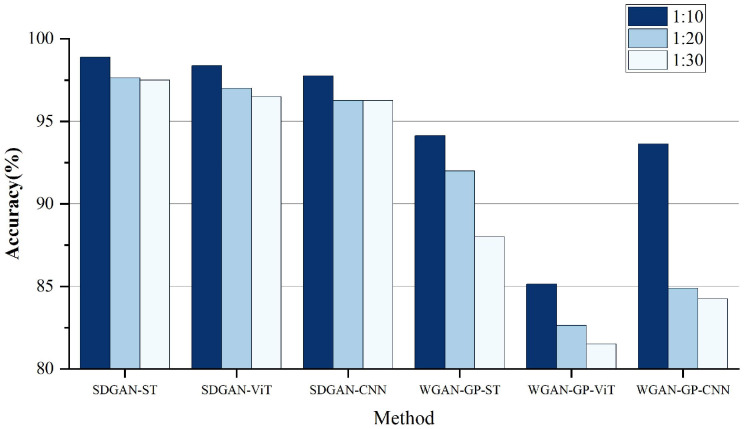
Diagnostic accuracy of various methods on CWRU dataset under three imbalanced ratios.

It should be noted that the validation and test sets are constructed in a balanced manner for fair model selection and performance evaluation. Therefore, the reported accuracy, together with the confusion matrices, can still provide a meaningful evaluation of the class-wise diagnostic performance under the current experimental setting. As is shown in Table 3 and Figure 4, our SDGAN-ST achieves the highest accuracy under various imbalanced ratios, reaching 98.88%, 97.63%, and 97.50%. As a comparison, those methods without data generation can only reach 84.75%, 71.25%, and 69.38% at most, reflecting the effectiveness of the SDGAN-ST for noisy imbalanced fault diagnosis. As the IR increases, the diagnostic accuracy of all methods gradually decreases. This decline can be attributed to the limited number of fault samples, which results in increased difficulty in training GAN and further leads to low-quality generated samples. This, in turn, negatively impacts the training and predictive capabilities of the classifiers.

**Figure 5:**
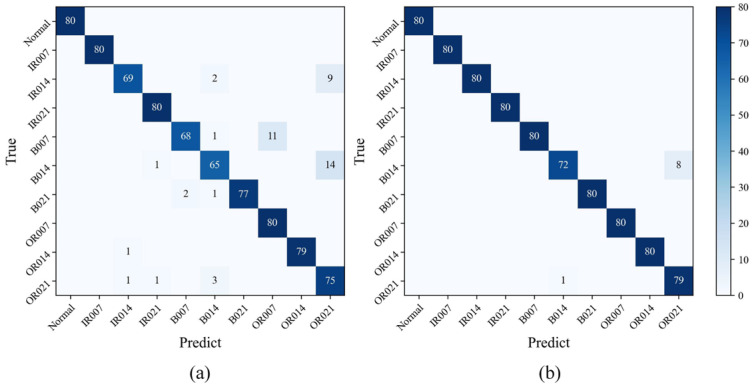
The confusion matrices diagnosed by two different methods in 1:10 IR and 20% NR: (**a**) ordinary WGAN-GP with Swin Transformer; and (**b**) SDGAN with Swin Transformer. The results show the class-wise diagnostic performance under noisy imbalanced conditions and demonstrate the ability of the proposed method to correctly identify different fault categories.

It is worth noting that all the diagnosis models with SDGAN have a higher accuracy than those with the ordinary WGAN-GP under the same IR. This result proves that the SDGAN exhibits strong anti-noise capability and effectively mitigates the negative impact of label noise. Furthermore, the confusion matrices presented in Figure 5 reveal that the ordinary WGAN-GP based diagnosis model exhibits low recall for certain classes. This deficiency can be attributed to mislabeled samples, which misguide the classifier and lead to the acquisition of incorrect knowledge. As a consequence, the label noise significantly impedes the classifier from improving its recognition capabilities for these classes. In contrast, the model utilizing the SDGAN achieves high accuracy across all classes.

It is also can be seen that the Swin Transformer always gets higher accuracy than the CNN and ViT. The classifiers based on the CNN utilize convolution kernels to extract features from local areas, limiting the receptive field of the kernel and potentially overlooking important pixels outside the operated region. On the other hand, the ViT leverages a self-attention mechanism, which enables it to consider all pixels in the input image and capture global information. But this makes ViT without any prior knowledge, and it is difficult to show the superiority of its global receptive field on small-scale datasets. However, the Swin Transformer still uses the self-attention in ViT as its core operation, while also using the local window in CNN, and the shifted window mechanism to maintain the global receptive field. Therefore, the Swin Transformer has a more stable and stronger ability in feature extraction and achieves a higher accuracy in image classification. However, the model using the WGAN-GP with Swin Transformer is still inferior to the model using the SDGAN with ViT or CNN, indicating that the improvement of the SDGAN-ST is mainly due to the SDGAN.

### 4.2. Case II: Evaluation on Real-World OCB Dataset

(1) Dataset Construction

**Figure 6:**
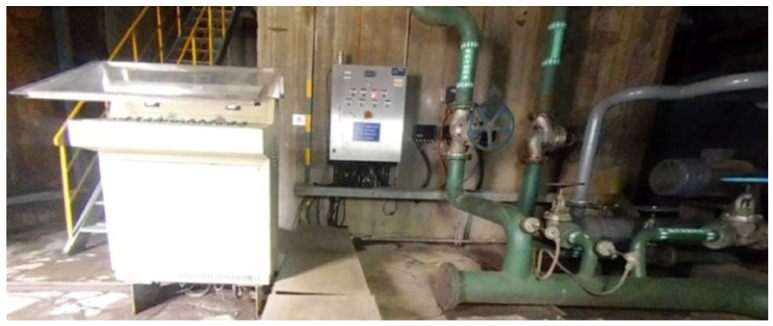
The oxygen compressor.

In this section, we employ a real-world oxygen compressor bearing (OCB) dataset to evaluate our SDGAN-ST. The oxygen compressor is from the largest smart factory for melting copper as shown in Figure 6. Its primary function is to consistently provide oxygen to a reaction tank. As the compressor operates, an acceleration sensor collects the vibration signals of the motor drive-end bearing per five seconds, forming the OCB dataset. This dataset contains three-class signals of normal condition, inner race fault and outer race fault generated during the compressor’s real-time operations.

With 1024 points as a signal and converted into an image, 400 samples can be obtained for each fault class. We select 300 normal condition samples and several fault samples to construct the OCB noisy imbalanced datasets under an IR of 1:10, 1:20, and 1:30 and an NR of 20%. To verify the ability to recall fault signals in the real world, each fault class in test set includes all 400 samples. To ensure that the test set is balanced, the normal condition class also contains 400 samples. More details are shown in Table 4.

(2) Data Generation

**Figure 7:**
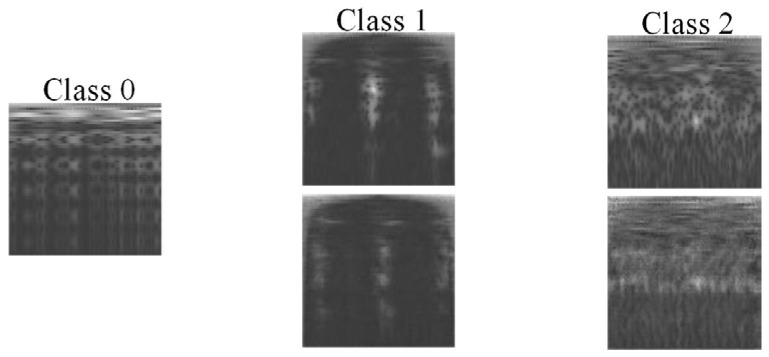
The data display of 3 classes on OCB balanced dataset. In a pair of images arranged up and down in class 1 and 2, the top one is real and the bottom one is generated. The figure shows that the generated samples preserve similar time-frequency patterns to the real samples.

In this case, the generators of class 1 and 2 are produced from the SDGAN. Subsequently, data generation is performed for both classes. Finally, the number of each fault class is same as the normal class and the balanced dataset is obtained. The balanced dataset in the IR of 1:10 is displayed in Figure 7.

(3) Noisy Imbalanced Fault Diagnosis

**Figure 8:**
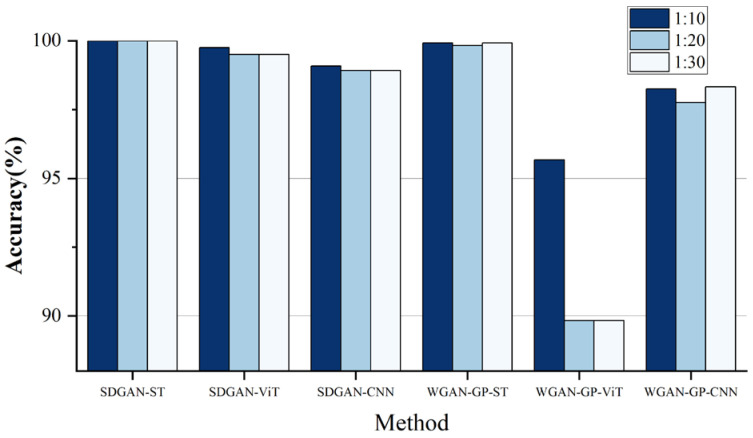
Diagnostic accuracy of various methods on OCB dataset under three imbalanced ratios.

The setup of comparison methods is the same as the Case I. And the experimental results on OCB noisy imbalanced dataset are shown in Table 5 and Figure 8. Since this case is a three-class problem and there are obvious differences among the different classes (shown in Figure 7), all the models can achieve relatively good results. Our SDGAN-ST reaches 100% under three IRs, which is the highest among the models. For the original imbalanced dataset without data generation, all methods can only achieve an accuracy of 33.33% or 66.67%, which can be explained that the imbalance of dataset during the training stage leads to the preference for a certain class.

### 4.3. The Anti-Noise Performance of SDGAN

In this part, the effect of the SDGAN is mainly focused. We construct three datasets with an NR of 20%, 30%, and 40% (IR is maintained as 1:10) on the CWRU and OCB datasets separately. The SDGAN and ordinary WGAN-GP are used as data generation models, respectively, with the Swin Transformer as a classifier to evaluate the anti-noise ability of SDGAN. The experimental results are shown in Table 6.

As the NR is increased, the accuracy of the ordinary WGAN-GP method decreases sharply, while that of the SDGAN remains at a high level. When the NR is 20%, the accuracy difference between the WGAN-GP and SDGAN methods is very small. This is because the neural network will pay more attention to the correctly labeled samples which appear frequently in the training set, and even the WGAN-GP without denoising capability is almost unaffected by mislabeled samples. As the amount of label noise grows, the WGAN-GP model becomes prone to learning incorrect information and synthesizing a large number of noisy samples. Consequently, this results in a significant drop in diagnostic accuracy. On the other hand, the SDGAN-based methods remain unaffected even when the NR is increased.

**Figure 9:**
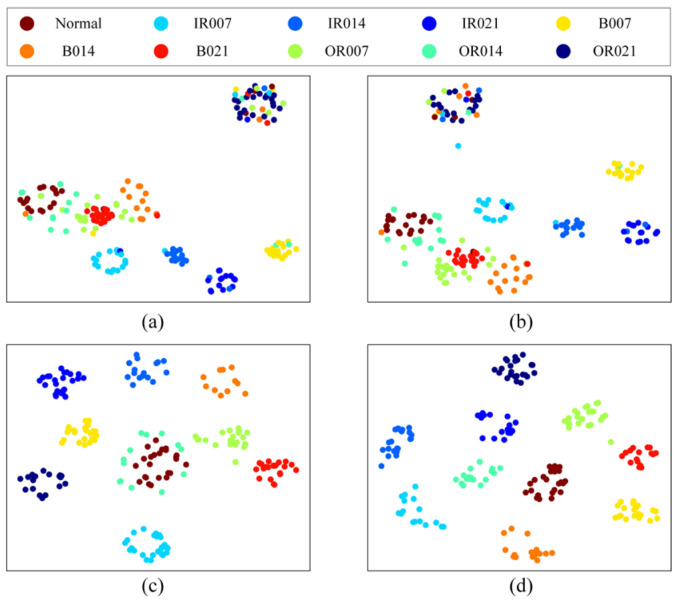
The sample distribution visualized by t-SNE on CWRU dataset in 1:10 IR and 20% NR: (**a**) original noisy imbalanced data; (**b**) generated data by ordinary WGAN-GP; (**c**) original data in safe domain; and (**d**) generated data by SDGAN. Different colors represent different fault categories, as indicated by the legend. The visualization illustrates the distribution relationship among original samples, safe-domain samples, and generated samples.

To observe the negative effect of label noise and the anti-noise performance of the SDGAN, the t-SNE technique is used to visualize the sample distribution during different stages. The input of t-SNE is 1D vectors obtained by flattening the samples (i.e., 2D images) in CWRU dataset. The label noise can be clearly observed in the original dataset, as depicted in Figure 9a. Consequently, when using the WGAN-GP, the generated dataset contains numerous mislabeled samples due to the limitations of the training set, as illustrated in Figure 9b. In contrast, Figure 9c represents the samples that have been selected from a safe domain, where there is minimal overlapping among classes. This indicates that the label noise has been effectively removed. As a result, SDGAN is able to successfully augment high-quality data for each fault class, as demonstrated in Figure 9d.

Although the SDGAN-ST achieves promising diagnostic performance, its computational cost should be considered in practical deployment. The proposed framework first converts 1D vibration signals into 2D time-frequency images using wavelet transform and then employs a Swin-Transformer-based classifier, which may introduce higher computational complexity than lightweight 1D diagnosis models. In addition, the GAN module increases the offline training cost. Developing lightweight time-frequency transformation strategies and compact classifiers for real-time industrial diagnosis will be investigated in future work.

## 5. Conclusions

This paper proposes the SDGAN-ST, a safe-domain generative adversarial network with a Swin Transformer, to address the imbalanced fault diagnosis with label noise. The SDGAN-ST comprises of a denoising module, a data generation module and a Swin-Transformer-based classifier. The denoising module utilizes a safe domain selection method to identify safe domains for each fault class. This ensures that only noise-free samples are retained while filtering out any noise. The samples in safe domains are not only beneficial to the convergence and generation quality of the generation model, but also to the high accuracy of the fault classifier. Additionally, we introduce the Swin Transformer as the classifier for diagnosing faults. Experimental results demonstrate that the SDGAN-ST achieves a high accuracy when applied to an imbalanced CWRU dataset with label noise, indicating its effectiveness in addressing noisy imbalanced fault diagnosis problems. Furthermore, we validate the effectiveness of the SDGAN-ST in real-world scenarios using the OCB dataset.

In the future, we will work on developing a more effective denoising method and combining it with GAN to achieve data generation for the imbalanced data with label noise in fault diagnosis. More advanced noisy-label learning strategies, such as co-teaching, robust loss functions, and self-supervised denoising, will be considered to further improve the robustness of the proposed framework. In addition, the sensitivity of key parameters such as the neighborhood size *K*, more comprehensive evaluation metrics for both diagnostic performance and generated sample quality, and lightweight model design for real-time industrial deployment will be further investigated.

## Figures and Tables

**Table 1:** Structure of Swin Transformer.

Layer Name		Output Shape	Attention Head Number
Patch Partition Module		(32,32,16)	-
Stage 1	Linear Embedding	(32,32,16)	-
	2 blocks	(32,32,16)	3
Stage 2	Patch Merging	(16,16,32)	
	2 blocks	(16,16,32)	6
Stage 3	Patch Merging	(8,8,64)	-
	2 blocks	(8,8,64)	12
Stage 4	Patch Merging	(4,4,128)	-
	2 blocks	(4,4,128)	24
MeanPooling		(1,128)	-
MLP Classification Head		(1,10)	-

**Table 2:** Description of CWRU noisy imbalanced datasets.

Dataset		Fault Diameter (mm)	Image Size	Training Set	Valid Set	Test Set	Label
Majority		Normal	128 × 128	300	20	80	0
Minority (1:10/1:20/1:30)	Inner Race	0.007	128 × 128	30(6 ^a^)/15(3)/10(2)	20	80	1
		0.014	128 × 128	30(6)/15(3)/10(2)	20	80	2
		0.021	128 × 128	30(6)/15(3)/10(2)	20	80	3
	Ball	0.007	128 × 128	30(6)/15(3)/10(2)	20	80	4
		0.014	128 × 128	30(6)/15(3)/10(2)	20	80	5
		0.021	128 × 128	30(6)/15(3)/10(2)	20	80	6
	Outer Race	0.007	128 × 128	30(6)/15(3)/10(2)	20	80	7
		0.014	128 × 128	30(6)/15(3)/10(2)	20	80	8
		0.021	128 × 128	30(6)/15(3)/10(2)	20	80	9

^a^ The number of samples with label noise.

**Table 3:** Accuracy of various methods on CWRU dataset.

Generation Model	Classifier	Imbalance Ratio		
		1:10	1:20	1:30
SDGAN	Swin Transformer	98.88 ^b^	97.63	97.50
	ViT	98.38	97.00	96.50
	CNN	97.75	96.25	96.25
WGAN-GP	Swin Transformer	94.13	92.00	88.00
	ViT	85.13	82.63	81.50
	CNN	93.63	84.88	84.25
--	Swin Transformer	84.75	71.25	69.38
	ViT	70.00	58.50	51.25
	CNN	81.38	66.13	60.50

^b^ The highest accuracy in 5 experiments (%).

**Table 4:** Description of OCB noisy imbalanced datasets.

Dataset		Image Size	Training Set	Test Set	Label
Majority	Normal	128 × 128	300	400	0
Minority (1:10/1:20/1:30)	Inner Race	128 × 128	30(6)/15(3)/10(2)	400	1
	Outer Race	128 × 128	30(6)/15(3)/10(2)3	400	2

**Table 5:** Accuracy of various methods on OCB dataset.

Generation Model	Classifier	Imbalance Ratio		
		1:10	1:20	1:30
SDGAN	Swin Transformer	100.00	100.00	100.00
	ViT	99.75	99.50	99.50
	CNN	99.08	98.92	98.92
WGAN-GP	Swin Transformer	99.92	99.83	99.92
	ViT	95.67	89.83	89.83
	CNN	98.25	97.75	98.33
--	Swin Transformer	66.67	66.67	33.33
	ViT	33.33	33.33	33.33
	CNN	33.33	33.33	33.33

**Table 6:** Diagnostic accuracy in different NRs on two datasets.

Method		SDGAN-ST	WGAN-GP-ST
CWRU	20%	98.88	94.13
	30%	97.75	90.38
	40%	97.75	88.38
OCB	20%	100	99.92
	30%	100	99.83
	40%	100	99.75

## Data Availability

The original contributions presented in this study are included in the article. Further inquiries can be directed to the corresponding author.
